# CCR5/CXCR3 antagonist TAK-779 prevents diffuse alveolar damage of the lung in the murine model of the acute respiratory distress syndrome

**DOI:** 10.3389/fphar.2024.1351655

**Published:** 2024-02-21

**Authors:** Aleksandr S. Chernov, Maksim V. Rodionov, Vitaly A. Kazakov, Karina A. Ivanova, Fedor A. Meshcheryakov, Anna A. Kudriaeva, Alexander G. Gabibov, Georgii B. Telegin, Alexey A. Belogurov

**Affiliations:** ^1^ Shemyakin and Ovchinnikov Institute of Bioorganic Chemistry, Russian Academy of Sciences, Moscow, Russia; ^2^ Medical Radiological Research Center (MRRC), A.F. Tsyb-Branch of the National Medical Radiological Research Center of the Ministry of Health of the Russian Federation, Moscow, Russia; ^3^ Department of Life Sciences, Higher School of Economics, Moscow, Russia; ^4^ Department of Chemistry, Lomonosov Moscow State University, Moscow, Russia; ^5^ Department of Biological Chemistry, Ministry of Health of Russian Federation, Russian University of Medicine, Moscow, Russia

**Keywords:** SARS-CoV-2, COVID-19, CCR5/CXCR3, TAK-779, diffuse alveolar damage of the lung, acute respiratory distress syndrome, macrophage inflammatory proteins, tocilizumab (IL-6 inhibitor)

## Abstract

**Introduction:** The acute respiratory distress syndrome (ARDS), secondary to viral pneumonitis, is one of the main causes of high mortality in patients with COVID-19 (novel coronavirus disease 2019)—ongoing SARS-CoV-2 infection— reached more than 0.7 billion registered cases.

**Methods:** Recently, we elaborated a non-surgical and reproducible method of the unilateral total diffuse alveolar damage (DAD) of the left lung in ICR mice–a publicly available imitation of the ARDS caused by SARS-CoV-2. Our data read that two C–C chemokine receptor 5 (CCR5) ligands, macrophage inflammatory proteins (MIPs) MIP-1α/CCL3 and MIP-1β/CCL4, are upregulated in this DAD model up to three orders of magnitude compared to the background level.

**Results:** Here, we showed that a nonpeptide compound TAK-779, an antagonist of CCR5/CXCR3, readily prevents DAD in the lung with a single injection of 2.5 mg/kg. Histological analysis revealed reduced peribronchial and perivascular mononuclear infiltration in the lung and mononuclear infiltration of the wall and lumen of the alveoli in the TAK-779-treated animals. Administration of TAK-779 decreased the 3–5-fold level of serum cytokines and chemokines in animals with DAD, including CCR5 ligands MIP-1α/β, MCP-1, and CCL5. Computed tomography revealed rapid recovery of the density and volume of the affected lung in TAK-779-treated animals.

**Discussion:** Our pre-clinical data suggest that TAK-779 is more effective than the administration of dexamethasone or the anti-IL6R therapeutic antibody tocilizumab, which brings novel therapeutic modality to TAK-779 and other CCR5 inhibitors for the treatment of virus-induced hyperinflammation syndromes, including COVID-19.

## Introduction

The socio-medical significance of the COVID-19 pandemic is hard to overestimate as it has led to the death of more than 6.9 million people around the world. The treatment of severe COVID-19, which is accompanied by the development of acute respiratory distress syndrome or diffuse alveolar damage (ARDS/DAD), is still challenging (Ntatsoulis et al., 2021). Clinical signs of ARDS include a dysfunction of the alveolar epithelium and erupted gas exchange. An attempt to restore the condition to normal often leads to a fibroproliferative state (Fears et al., 2022). The ARDS causes disability, morbidity, and mortality, while the current treatment is focused on basial patient support to give the lungs enough time to recover (Luo et al., 2020; Wick et al., 2021). The course of ARDS is generally associated with a cytokine storm, consisting in the enormous release of cytokines and chemokines (Martonik et al., 2023).

The immune response toward SARS-CoV-2 in humans occurs in several stages. Activation of the type I interferon (IFN I) response induces the recruitment of macrophages, NK cells, and neutrophils to the virus penetration site (Tamir et al., 2022). It is believed that resident alveolar macrophages are the first defensive line to fight the virus (Kumar et al., 2023), and neutrophils may undergo TNF-dependent necroptosis among seriously ill patients (Schweizer et al., 2021; Mairpady Shambat et al., 2022). During the adaptive immune response stage, CD4^+^ and CD8^+^ T cells spread and hyperactivate, which may lead to a lack of reactivity or cell death (Alberca and Baddour, 2021). In addition, immune response modulation is actively driven by Th17 and Th22 subpopulations (Martonik et al., 2023). The levels of IgG, IgM, and IgA antibodies produced by B lymphocytes increase during COVID-19 infection (Ruhl et al., 2021). B cells also release IL-6, which intensifies the cytokine storm (Upasani et al., 2021). As a result of cytokine release, the integrity of endotheliocytes is erupted and TxA2 is released, which, in turn, causes thrombosis (Conti et al., 2021). The physiological effect of cytokines is maintained mainly through the activation of the JAK/STAT signaling pathway. Levels of cytokines, e.g., IL-6, positively correlate with the mortality of COVID-19 patients (Stebbing et al., 2021). Fanning et al. showed that the level of C–C/C–X–C chemokine ligands and C-reactive protein (CRP) are correlated with COVID-19 progression in patients treated by convalescent donor plasma (Fanning et al., 2021). As the cytokine storm escalates, apoptosis of the pulmonary epithelium increases, the blood–air barrier and vessels become damaged, resulting in alveolar edema and hypoxia (Martonik et al., 2023). A high level of cytokines in the blood is also associated with bacterial superinfections in COVID-19 patients (Mairpady Shambat et al., 2022).

The current list of Food and Drug Administration (FDA)-approved COVID-19 therapeutics includes the anti-interleukin-6 receptor monoclonal antibody Actemra (tocilizumab) (Abani et al., 2021; Salama et al., 2021), the antiviral nucleotide analog Veklury (remdesivir) (Beigel et al., 2020), the inhibitor of Janus kinases JAK1 and JAK2 Olumiant (baricitinib) (Kalil et al., 2021; Marconi et al., 2021), and a mixture of the 3CLpro protease inhibitor and inhibitor of HIV-1 protease CYP3A Paxlovid (nirmatrelvir and ritonavir) (Hammond et al., 2022). Therapy for coronavirus infection evolved in various directions. For example, remdesivir suppresses coronavirus replication by inhibiting RNA-dependent RNA polymerase (Martonik et al., 2023). Another approach to the COVID-19 treatment is the administration of glucocorticosteroids. These immunosuppressors modify gene expression and trigger the synthesis of NF-kB inhibitors, reducing the production of IL-1 and IL-6 (Martonik et al., 2023). Treatment of COVID-19 patients was accomplished by the monoclonal antibody olokizumab, which has high affinity to IL-6 and neutralizes this cytokine (Alfinito et al., 2020). Furthermore, the anti-IL-17 monoclonal antibody netakimab, utilized for patients with psoriasis and ankylosing spondylitis, may be used for COVID-19 treatment (Bryushkova et al., 2022). Pharmacodynamically, the netakimab effect is beneficial since IL-17 increases the level of inflammatory mediators such as G-CSF, IL-6, IL-1β, TNFα, IL-8, and matrix metalloproteases. Tocilizumab proved its clinical effectiveness in patients during the coronavirus pandemic (Io et al., 2021). One more approach of the treatment is using colchicine, an indirect IL-6 inhibitor, which is also applied in the treatment of coronary heart disease (Bonifácio et al., 2023). Colchicine suppresses the recruitment of neutrophils, inhibits cytoskeleton metabolism, and opposes SARS-CoV-2 functionality in human cells (Kasiri et al., 2023). Rodriguez et al. suggested IL-8 antagonists to be prospective agents to treat severe coronavirus infection (Rodriguez et al., 2021). Indeed, convalescent plasma (Misset et al., 2023) and SARS-CoV-2 neutralizing antibodies (Chen et al., 2021; Guo et al., 2021) may also be regarded as direct agents for virus clearance.

Another pharmacological group, JAK inhibitors, such as tofacitinib, seems to be also the perspective (Sharma and Ali, 2021). Baricitinib, a JAK1/JAK2 inhibitor, reduces the content of phosphorylated STAT proteins, which prevents the proinflammatory effects of IL-6, IL-12, IL-23, and IFN-γ (Bryushkova et al., 2022). It is also suggested that JAK-STAT pathway inhibition may ameliorate the condition during bacterial sepsis-induced ARDS (Batra et al., 2022). The MAPK, RAS, and PI3K-AKT pathways may be potential targets in ARDS as well (Batra et al., 2022). Metformin, used to treat type 2 diabetes, is also considered a promising drug that acts at different stages of the SARS-CoV-2 development (viral entry, viral replication, etc.), but it has yet to be investigated (Varghese et al., 2021). Additionally, COVID-19 treatment can be carried out by HIF pathway exposure. Thus, preclinical trials of FG-4592 (roxadustat) that suppressed PHDs were conducted. The drug activated HIFs and reduced viral burden and respiratory symptoms on the fourth day after infection (Wing et al., 2022). Ewart et al. showed that small-molecule acylguanidine BIT225 prevents weight loss in SARS-CoV-2-infected K18 mice, suppresses virus reproduction, and exhibits anti-inflammatory properties (Ewart et al., 2023).

Complement inhibitors may also be used in the treatment of COVID-19. During coronavirus infection, C5a interacts with its C5aR1 receptor, which promotes the migration of monocytes/macrophages and neutrophils into the lung tissue resulting in the cytokine storm (Chouaki Benmansour et al., 2021). The administration of the anti-C5a monoclonal antibody vilobelimab to COVID-19 patients showed a promising therapeutic effect (Vlaar et al., 2020). A number of C5 inhibitors, such as the antibody eculizumab and peptide zilucoplan, preventing the production of C5b-9, showed beneficial results in clinical trials (Burwick et al., 2022; De Leeuw et al., 2022). The pharmacological effect of compstatin AMY–101, an inhibitor of C3, was recently reported (Mastaglio et al., 2020; Skendros et al., 2022). Finally, drugs, which target the lectin pathway of complement, such as the human anti-MASP-2 antibody narsoplimab, are satisfactorily applied in the coronavirus infection management since all COVID-19 patients receiving narsoplimab achieved recovery and survived (Rambaldi et al., 2020).

Summarizing, the global medical community is faced with creating and introducing into clinical practice new effective and safe methods of treating not only COVID-19 but also similar virus-induced lung inflammation syndromes. Despite the fact that SARS-CoV-2 lost its pandemic status, the search for systemic and etiotropic treatment of ARDS should prepare humanity for possible future viral epidemics. The chemokine-driven migration of activated T cells and monocytes to the infection site is critical for the progression of COVID-19 (Gamage et al., 2020; Çelik et al., 2023). Several reports showed that C–C/C–X–C chemokine ligands CCL3, CCL4, and CXCL-10 take part in the development of SARS-CoV-2 infection (Singh et al., 2021; Calvier et al., 2023; Martonik et al., 2023). It is known that in the severe form of the disease, CXCR2 signaling is activated, while in the favorable course of the disease, a T helper “Th1–Th17” profile, marked by an upregulation of the CXCR3 pathway activator genes, is observed (Rodriguez et al., 2021). Recently, Dai et al. showed that H5N1 AIV-induced inflammatory lung injury is driven by infiltrating inflammatory macrophages with massive viral replication and an emerging interaction of cell populations through various chemokines, including CCL4 (Dai et al., 2023). Thus, a possible way to treat coronavirus infection is to target chemokine receptors. Among the three anti-chemokine drugs (leronlimab, maraviroc, and cenicriviroc), the latter seems to be the most optimal since it, by inhibiting CCR2 and CCR5 pathways, precludes the pulmonary and vascular sequelae associated with COVID-19 (Files et al., 2022).

TAK-779 is also a potent and selective nonpeptide antagonist of CCR5/CXCR3 (Gao et al., 2003), with a K_i_ value of 1.1 nM. The CCR5-related cognate ligands include CCL3, CCL4 (also known as MIP 1α and 1β, respectively), and CCL3L1. CCR5 also interacts with CCL5 (a chemotactic cytokine protein, also known as RANTES). TAK-779 effectively and selectively inhibits R5 HIV-1 in MAGI-CCR5 cells with an EC_50_ value of 1.2 nM (Baba et al., 1999). In the dosage of 10 mg/kg per day, TAK-779 significantly prolongs the allograft survival of the rat intestinal transplantation model. It inhibits the migration of T cells but not its proliferation. TAK-779 also decreases the number of CD4^+^ and CD8^+^ T cells in the spleen, blood, and recipient mesenteric lymph nodes (MLNs) (Takama et al., 2011). Zhu et al. demonstrated that TAK-779 in the dosage of 150 µg per mouse suppresses the development of experimental autoimmune encephalomyelitis (EAE) in myelin oligodendrocyte glycoprotein (MOG)-immunized C57BL/6 mice. It decreases the infiltration of CXCR3- and CCR5-bearing leukocytes into the spinal cord (Ni et al., 2009). TAK-779 ameliorates pulmonary granulomatosis in C57BL/6 mice and diminishes the pool of CXCR3^+^CD4^+^ and CCR5^+^CD4^+^ T lymphocytes in the bronchoalveolar lavage fluid (Kishi et al., 2011). Tokuyama et al. showed that TAK-779 decreases the recruitment of monocytes/macrophages, thereby precluding dextran sodium sulfate-induced colitis in C57BL/6 mice (Tokuyama et al., 2005). TAK-779 also impedes the progression of chronic vasculopathy, fibrosis, and cellular infiltration by reducing the number of CD4-, CD8-, and CD11c-positive cells recruited to the transplanted allografts (Akashi et al., 2005). Furthermore, it was shown that in a Pan02 murine tumor model, a TAK-779-induced disruption of the CCR5/CCL5 pathway led to decreased Treg migration to the tumor (Tan et al., 2009). Likewise, TAK-779 inhibits the homing of microglia in response to scrapie infection *in vitro* and *in vivo* (Marella and Chabry, 2004).

The main restriction of COVID-19 modeling in mice is that SARS-CoV-2 does not bind to mouse ACE2 (mACE2) (Rawle et al., 2021). Thus, various artificial models are used to study the effects of coronavirus infection. Yinda et al. reported that the administration of 10^4^ TCID_50_ or 10^5^ TCID_50_ SARS-CoV-2 led to 80% and absolute lethality, respectively, in K18-hACE2 mice, as well as a mild form of the disease in mice that received 10^2^ TCID_50_ SARS-CoV-2 (Yinda et al., 2021). Nevertheless, the disadvantage of this model is the possible fatal outcome after a fulminant SARS-CoV-2 brain infection (Kumari et al., 2021; Bishop et al., 2022). Two additional major obstacles of animal models, involving SARS-CoV-2 inoculation, are the absence of prolonged monitoring due to the high mortality rate and generally high variability between individual animals in terms of cytokine levels and other immunological indicators. These features limit statistical analysis and yield a binary “yes or no” result of the experimental therapy. Here, we assessed the therapeutic effects of dexamethasone, tocilizumab, and TAK-779 on the course of the ARDS in the non-lethal, highly reproducible ICR DAD murine model mimicking SARS-CoV-2 infection (Chernov et al., 2022) by histological evaluation, cytokine profiling, and computed tomography analysis.

## Materials and methods

### Ethics statement

ICR male mice in the SPF category with an average weight of 37.4 ± 1.4 g were used. All animals were housed under standard conditions in the animal breeding facility of BIBCh RAS (the Unique Research Unit Bio-Model of the IBCh RAS; the Bioresource Collection–Collection of SPF-Laboratory Rodents for Fundamental, Biomedical, and Pharmacological Studies, Сontract 075-15-2021-1067). All experiments and manipulations with animals were approved by the Institutional Animal Care and Use Committee (IACUC № 831/22 from 12/04/22).

### Reagents

LPS (*Salmonella enterica*) was purchased from Millipore Sigma (United States), α-galactosylceramide was obtained from Avanti Polar Lipids (United States), propofol was obtained from Hana Pharmaceutical, Co. Ltd. (Republic of Korea), TAK-779 was obtained from MedChemExpress (United States), and tocilizumab was obtained from Roche (Japan).

### DAD modeling in ICR mice

DAD was induced by a single instillation into the left lung of the mixture consisting of 100 μL (1 mg/mL) LPS from *S. enterica* and 100 μL (50 μg/mL) α-galactosylceramide using a 20G intravenous catheter. As premedication, propofol at a dose of 20 mg/kg was intravenously injected for anesthesia shortly before the intubation of trachea.

### Administration and doses of test substances

The animals were randomly assigned to five groups with 20 animals in each group: (1)–control, intact animals; (2) intravenous injection of physiological saline (200 µL 0.9% NaCl) as a placebo during DAD induction; (3) intravenous injection of tocilizumab (25 mg/kg) during DAD induction; (4) intravenous injection of TAK-779 (2.5 mg/kg) during DAD induction; and (5) intravenous injection of dexamethasone (0.5 mg/kg) during DAD induction. Animals were monitored daily for 45 days.

### Computed tomography of the lungs

A CT scanner, MRS*CT/PET (MR Solution, United Kingdom), was used with following parameters: energy 40 kVp, exposure 100 ms, current 1 mA, and stepping angle 1°. During imaging, the animals were anesthetized with a 2% mixture of isoflurane and air at a temperature of +37°С. The CT images were processed using VivoQuant software (Invicro, United Kingdom). The lung volume and the average density of left (exposed) and right (control) lungs were measured in the automatic mode (using −400 to 100 Hounsfield units as the cut-off density). The volume was expressed in mm^3^, and the density, in Hounsfield units (HU).

### Plasma chemokine and cytokine measurement

Mouse blood for EDTA plasma was collected after 3 h post-DAD induction. Bio-Plex Pro Mouse Cytokine Panel 33-Plex (Bio-Rad, United States) was used to measure the levels of chemokines and cytokines. Plasma samples diluted at a ratio of 1:3 (50 μL) were incubated with magnetic beads, washed up, and then incubated with detecting antibodies and SA-PE, according to the manufacturer’s instructions. Data were obtained using the Luminex 200 analyzer and analyzed using xPONENT software.

### Histological examination

Five animals from each group were sacrificed at days 7 and 45, and histological studies were performed, as described previously (Sharapova et al., 2021; Chernov et al., 2022). In brief, the lungs were filled with a 10% solution of neutral formalin, embedded in paraffin, and 4–5-μm-width sections were stained with hematoxylin and eosin. The degree of fibrosis was examined on histological preparations stained by the Mallory method. Histological analysis of the lungs assessed the following morphological signs: peribronchial and perivascular mononuclear infiltration, infiltration of the walls and lumen of the alveoli by mononuclears, atelectasis, the presence or absence of necrosis foci, and level of fibrosis. The severity of various inflammatory phenomena in the lungs and the degree of pneumofibrosis were evaluated by a semi-quantitative method (in points), according to the 5-score scale (Sharapova et al., 2021). The Kernogan index in the blood vessels of the left lobe of the lungs was evaluated as the ratio of the thickness of the vascular wall to the radius of the vessel lumen as an important indicator of the throughput of the microcirculatory bed of the small circulatory circle using ZEN 2.6 lite software (Carl Zeiss, Germany).

### Statistical analysis

Data are presented as mean ± standard deviation. Differences between treatment and control groups were tested for significance using SigmaPlot software (SYSTAT Software Inc., Berkshire, United Kingdom), using Student’s t‐test. The *p*-values less than 0.05 were considered statistically significant.

## Results

### Treatment by TAK-779 significantly reduces mononuclear infiltration in the lung in the murine model of the SARS-CoV-2-related acute respiratory distress syndrome

**FIGURE 1:**
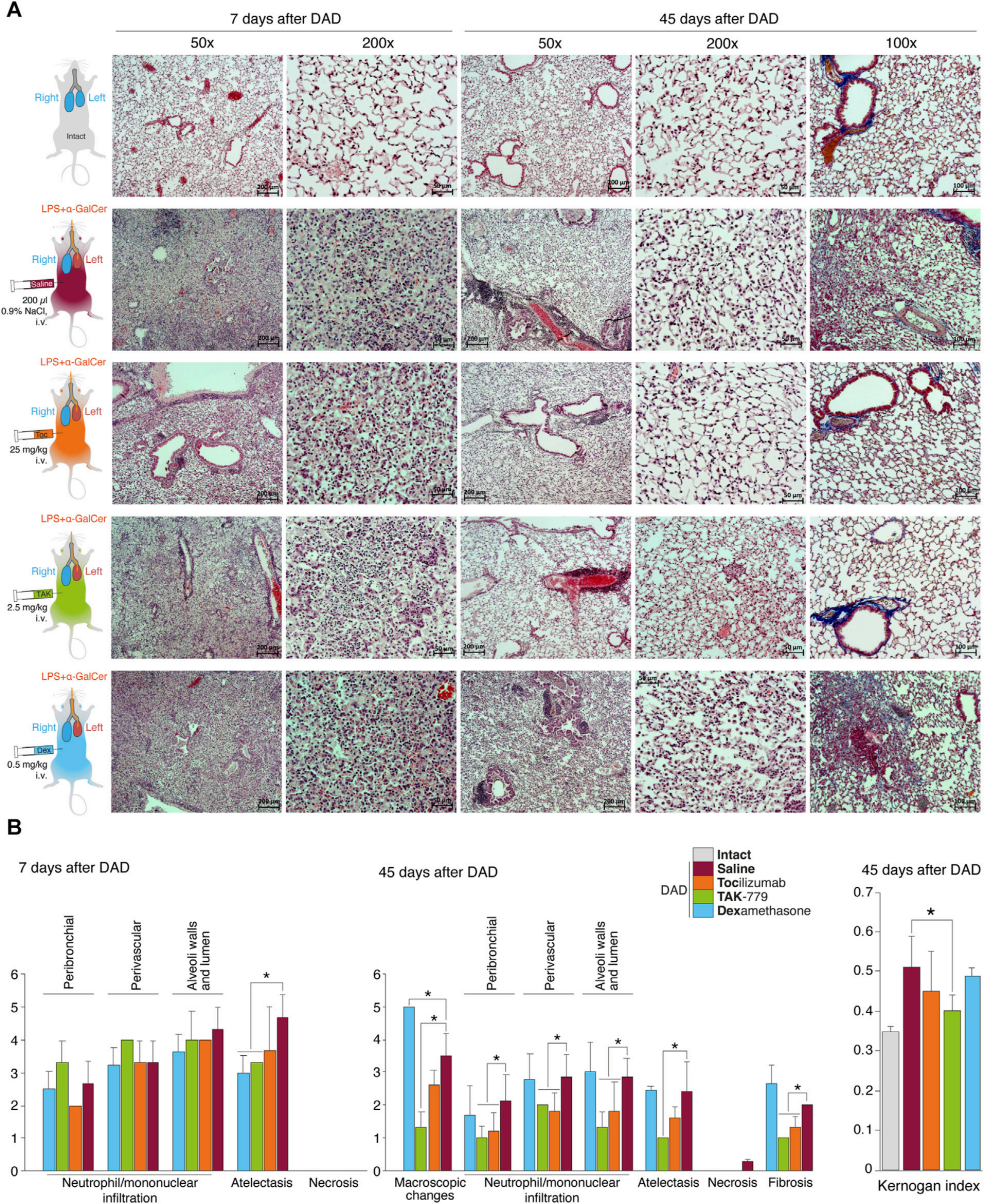
Reduced peribronchial and perivascular mononuclear infiltration in the lung and mononuclear infiltration of the wall and lumen of the alveoli in the TAK-779-treated ICR mice with DAD. **(A)** Histological analysis of the left lobe of the lungs of ICR mice with DAD on days 7th and 45th in comparison with unexposed animals (gray) and mice treated by saline (control, red), tocilizumab (orange), TAK-779 (green), and dexamethasone (blue). Stained with hematoxylin and eosin and by the Mallory method (right column). Magnification ×50, ×100, and ×200. **(B)** Semi-quantitative score (0–5 points) of peribronchial and perivascular mononuclear infiltration, mononuclear infiltration into the wall, and lumen of the alveoli; atelectasis; necrosis; and fibrosis on days 7 and 45 after DAD induction in unexposed mice (intact, gray), untreated mice (saline, red), and mice with DAD treated by tocilizumab (orange), TAK-779 (green), and dexamethasone (blue). The Kernogan index after 45 days after DAD induction in test groups is shown in the right side. Bars represent standard deviation. The statistically significant difference with non-treated animals with DAD (saline, red) is marked by an asterisk.

The DAD was induced in ICR mice by a single instillation into the left lung of the mixture consisting of LPS from *S. enterica* and α-galactosylceramide. On day 7 of monitoring, a similar pathomorphological finding was observed in all test groups: total or subtotal atelectasis of the left lobe with pronounced neutrophilic and mononuclear infiltration into the walls and lumen of the alveoli and moderate focal peribronchial and perivascular infiltration (Figure 1A). The right lobes were intact in all cases.

The degree of atelectasis of the left lobe of the lungs was minimal in the group of animals with DAD receiving dexamethasone and maximal in case of saline administration (placebo) (Figure 1B; Table 1). In animals treated with dexamethasone, the degree of neutrophil/mononuclear infiltration of the walls and lumen of the alveoli was estimated at 3.6 points; focal perivascular neutrophil/mononuclear infiltration corresponded to 3.3 points (Figure 1B; Table 1). The degree of focal peribronchial neutrophil and mononuclear infiltration was minimal among animals receiving tocilizumab and maximal among animals receiving TAK-779 (Figure 1B; Table 1). Focal perivascular neutrophil/mononuclear infiltration was also the highest in the TAK-779-treated group (Figure 1B; Table 1). Statistically significant differences between treated and non-treated animals were observed in the rate of atelectasis in groups of mice receiving TAK-779 and tocilizumab.

**TABLE 1:** Left lung damage score on day 7 after DAD induction in ICR mice treated with saline, tocilizumab, ТАК-779, and dexamethasone. The statistically significant difference with non-treated animals (saline) is marked by an asterisk.

Test group	Mononuclear infiltration			Atelectasis	Necrosis
	Peribronchial	Perivascular	Walls and lumen of the alveoli		
Intact	0	0	0	0	0
DAD + saline	2.7 ± 0.6	3.3 ± 0.6	4.3 ± 0.6	4.7 ± 0.6	0
DAD + tocilizumab	2.0 ± 0	3.3 ± 0.6	4.0 ± 0	3.7 ± 1.2	0
DAD + ТАК-779	3.3 ± 0.6	4.0 ± 0	4.0 ± 0.8	3.3 ± 0*	0
DAD + dexamethasone	2.5 ± 0.5	3.3 ± 0.5	3.6 ± 0.5	3.0 ± 0.5*	0

Total lesion of the left lobe with a significant reduction in its volume and total hypoventilation in all tested groups was observed 45 days after DAD induction (Figure 1A). Moderate focal perivascular and insignificant focal peribronchial mononuclear infiltration were detected. The walls and lumen of the alveoli were also diffusely infiltrated by mononuclear cells (Figure 1B; Table 2). The lumen of a significant part of the bronchi was partially or completely obstructed. Mononuclear infiltration of the walls and lumen of the alveoli, as well as signs of hypoventilation in case of dexamethasone administration, showed negative dynamics in comparison with the non-treated group (Figure 1B; Table 2). The latter was confirmed by the average estimated score of macroscopic changes (5.0) in the left lobe of the animals receiving dexamethasone (Figure 1B; Table 2). The fibrous changes in the left lobe also had a negative trend and were estimated at 2.7 points. The Kernogan index of dexamethasone-treated mice was comparable with the group of animals receiving saline (0.49 ± 0.02) (Figure 1B).

**TABLE 2:** Left lung damage score on day 45 after DAD induction in ICR mice treated with saline, tocilizumab, ТАК-779, and dexamethasone. The statistically significant difference with non-treated animals (saline) is marked by an asterisk.

Test group	Mononuclear infiltration			Macroscopic change	Atelectasis	Necrosis	Fibrosis
	Peribronchial	Perivascular	Walls and lumen of the alveoli				
Intact	0	0	0	0	0	0	0
DAD + saline	2.1 ± 0.7	2.9 ± 0.6	2.9 ± 0.5	3.5 ± 0.6	2.4 ± 0.8	0.29 ± 0.08	2.0 ± 0
DAD + tocilizumab	1.2 ± 0.5*	1.8 ± 0.5*	1.8 ± 0.8*	2.6 ± 0.4	1.6 ± 0.3	0	1.3 ± 0.3*
DAD + ТАК-779	1.0 ± 0.3*	2.0 ± 0*	1.3 ± 0.4*	1.3 ± 0.4*	1.0 ± 0*	0	1.0 ± 0*
DAD + dexamethasone	1.7 ± 0.8	2.8 ± 0.7	3.0 ± 0.8	5.0 ± 0*	2.5 ± 0.1	0	2.7 ± 0.5

Administration of tocilizumab resulted in significant improvement in the pathomorphological findings on day 45 of the observation in the left lobe of the lungs for all key indicators (Figure 1B; Table 2). The average score of mononuclear infiltration of the walls and lumen of the alveoli was estimated at 1.8 points, and focal peribronchial and perivascular mononuclear infiltration were estimated at 1.2 and 1.8 points, respectively. In addition, the left lobe of the lungs participated in gas exchange as areas of atelectasis, and fibrous changes were estimated on average at 1.6 (macroscopic score–2.6) and 1.3 points, respectively (Table 2). The Kernogan index evaluated in the group treated by tocilizumab had more favorable values (0.45 ± 0.10) compared to groups of animals treated with saline and dexamethasone (Figure 1B).

The most pronounced improvement on day 45 of the observation was detected in the tested group treated by TAK-779 (Figure 1B; Table 2). Macroscopic changes in the left lobe of the lungs corresponded to 1.3. Atelectasis, according to histological data, was estimated as 1.0. The degree of mononuclear infiltration of the walls and lumen of the alveoli was determined at 1.3 points, while focal peribronchial and perivascular mononuclear infiltration were determined at 1.0 and 2.0 points, respectively. The degree of fibrous changes in the left lobe was 1.0 points. The Kernogan index was also the most favorable in comparison with all the analyzed groups–0.40 ± 0.04 (Figure 1B).

### Treatment by TAK-779 suppresses the development of the cytokine storm in the murine model of the SARS-CoV-2-related acute respiratory distress syndrome

**FIGURE 2:**
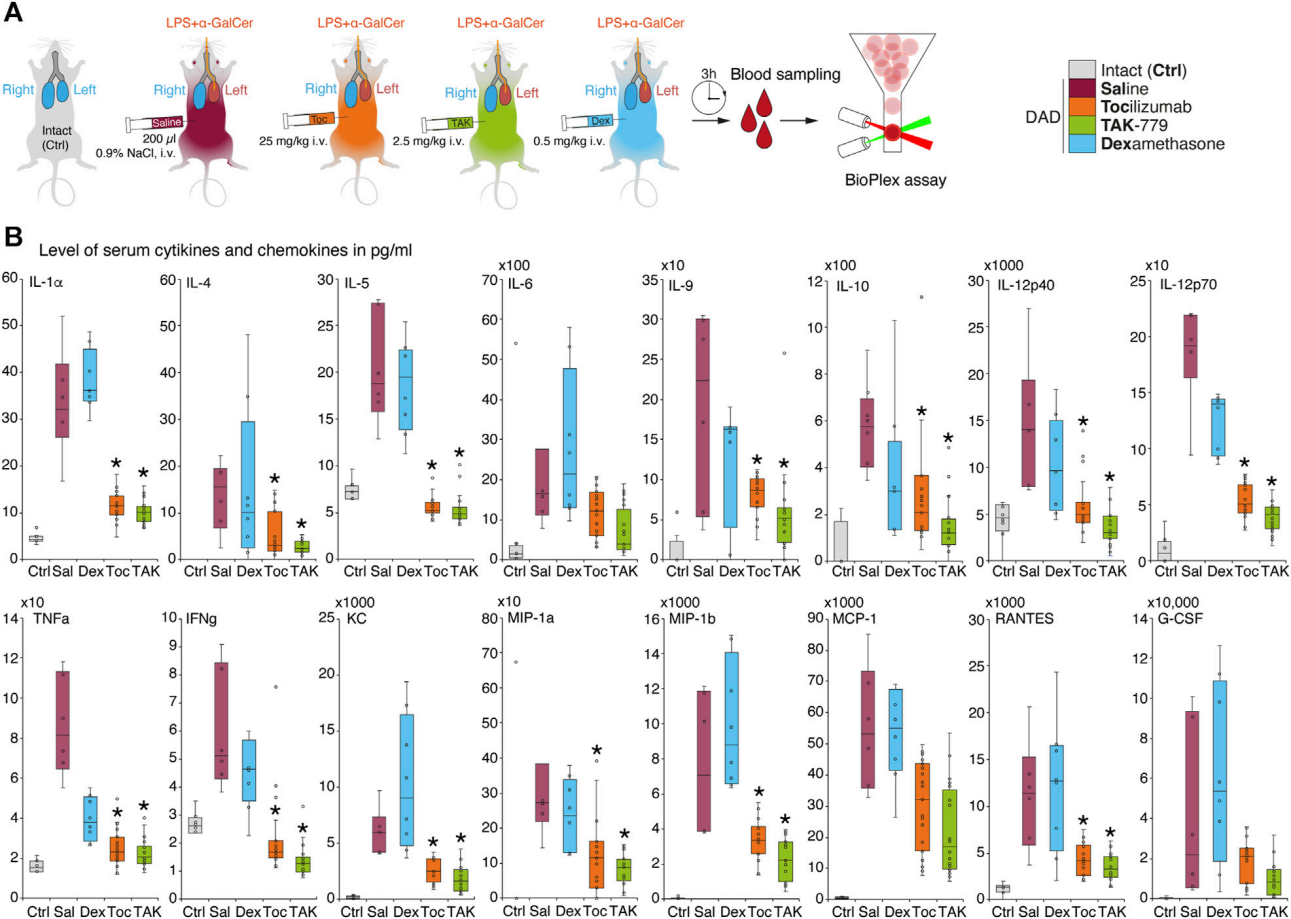
Administration of TAK-779 decreases 3–5-fold level of serum cytokines and chemokines in ICR mice with DAD. **(A)** Study design: blood was collected after 3 h post-DAD induction, and levels of cytokines and chemokines were estimated by multiplex immunoassay. **(B)** Levels of plasma cytokines and chemokines (pg/mL) in ICR mice 3 h after DAD induction treated by tocilizumab (orange), TAK-779 (green), and dexamethasone (blue) in comparison with unexposed (gray) and non-treated animals with DAD (red). Bars represent the median, interquartile range with standard deviation. Granulocyte colony-stimulating factor (G-CSF), keratinocyte chemoattractant (KC), tumor necrosis factor (TNF), interferon gamma (IFNγ), Regulated on Activation, Normal T-cell Expressed and Secreted (RANTES/CCL5), monocyte chemoattractant protein 1 (MCP-1/CCL2), and macrophage inflammatory proteins (MIP)–MIP-1α and MIP-1β. The statistically significant difference with non-treated animals with DAD (saline, red) is marked by an asterisk.

We next measured cytokine profiles in treated and non-treated ICR mice with induced DAD and compared it with unexposed animals (Figure 2A). Our data suggest that during the first 3 h, almost all cytokines and chemokines were significantly elevated in the plasma of mice with induced DAD compared to intact animals (Figure 2B). Levels of IL-5, IL-12p40, tumor necrosis factor (TNF), and interferon-gamma (IFNγ) were increased two- to five-fold; level of IL-1a, IL-6, IL-9, IL-10, IL-12p70 and RANTES/CCL5 was upregulated up to 10 times; and levels of IL-4, monocyte chemoattractant protein 1 (MCP-1/CCL2, chemokine C–C motif ligand 2), the keratinocyte chemoattractant (KC), and granulocyte colony-stimulating factor (G-CSF) were elevated up to two orders of magnitude in comparison with unexposed animals. Two CCR5 ligands macrophage inflammatory proteins (MIP), namely, (MIP-1α/CCL3) and (MIP-1β/CCL4) were upregulated up to three orders of magnitude compared to the background level. Administration of dexamethasone did not alter cytokine and chemokine profile mice with DAD except the level of TNF, which decreased twice in comparison with untreated animals. Injection of tocilizumab and TAK-779 resulted in similar, 3–5-fold downregulation of majority of cytokines except IL-6, MCP-1, and G-CSF (Figure 2B).

### Treatment by TAK-779 prevents collapse of the injured lung in the murine model of the SARS-CoV-2-related acute respiratory distress syndrome

**FIGURE 3:**
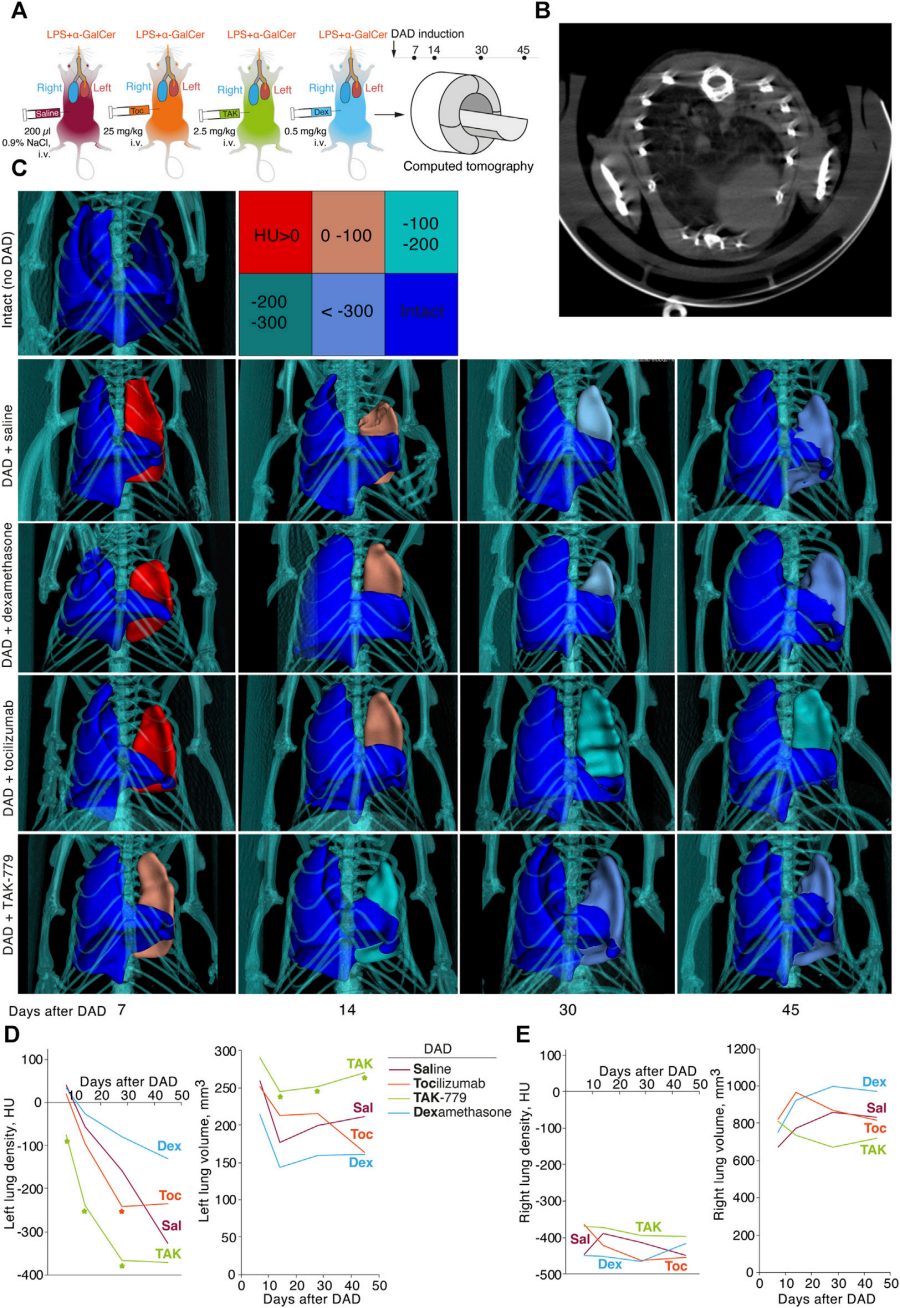
Treatment by TAK-779 induces fast recovery of the injured lung in ICR mice with DAD. **(A)** Study design: computed tomography (CT) was performed after days 7, 14, 30, and 45 after DAD induction. **(B)** Representative CT visualization of mouse lungs after DAD induction. Subtotal consolidation of the lung volume, less often mosaic-located areas of ground-glass opacities, alveolar consolidation, and rare areas of the intact lung tissue were observed. **(C)** Representative 3D CT reconstruction of the murine lungs from different groups. The color legend for Hounsfield units is shown. Density (HU) and volume (mm^3^) of murine left **(D)** and right **(E)** lungs of non-treated (Sal, red) and tocilizumab- (Toc, orange), TAK-779- (TAK, green), and dexamethasone (Dex, blue)-treated animals. The statistically significant difference with non-treated animals with DAD is marked by an asterisk.

A dynamic assessment of the volume of pulmonary lobes and its average density in Hounsfield units of the ICR mice from test groups was performed using a CT scanner, MRS*CT/PET, on days 7, 14, 30, and 45 after DAD induction (Figure 3A). Generally, we observed development of the specific pattern in the left lung in all groups: total or subtotal consolidation of the lung volume, less often mosaic-located areas of ground-glass opacities, alveolar consolidation, and rare areas of the intact lung tissue (Figure 3B).

Seven days after DAD induction, densities of the affected left lung of the mice treated by saline, tocilizumab and dexamethasone were 40.0 HU, 20.4 HU, and 33.4 HU, respectively (Figure 3С, D). In mice treated by TAK-779, the density of the left lung was significantly lower and equaled to −75 HU (Figures 3C, D). The volume of the affected lung 14 days after DAD induction decreased to less than 40% in the group of mice treated by TAK-779, whereas non-treated animals and dexamethasone-treated mice showed 60%–70% collapse of the left lung volume. Further monitoring of the density and volume of the affected lung in test groups revealed rapid recovery of the injured lung in TAK-779-treated mice, a minimally beneficial effect of tocilizumab, and a negative influence of dexamethasone administration. Treatment by TAK-779 first restored the caudal segments of the lung and further normalized the segments of the apex. Parameters of the non-affected right lung of mice from all test groups (Figure 3E) were equal to those of the intact mice.

## Discussion

In majority of clinical cases, innate and adaptive immunity overcomes SARS-CoV-2 infection; however, some patients develop a severe late stage, which promotes the cytokine storm and accelerates the deterioration of patients to the ARDS/DAD (Rondovic et al., 2022). The main pathological effect may be caused by hypersensitivity reactions rather than the virus itself, as Zhenfei et al. reported that formaldehyde-inactivated SARS-CoV-2 induces ARDS in human ACE2-transgenic mice (Bi et al., 2021).

Dexamethasone has evident anti-inflammatory effects and is widely used as an auxiliary treatment for viral pneumonia (Andreakos et al., 2021). Low and moderate doses of dexamethasone decrease the mortality rate in patients with a severe form of COVID-19. However, it is not recommended for patients with mild symptoms (Ahmed and Hassan, 2020). In patients hospitalized with COVID-19, the use of dexamethasone resulted in lower 28-day mortality among those who were receiving either invasive mechanical ventilation or oxygen alone but not among those receiving no respiratory support (Horby et al., 2021). Interestingly, it was demonstrated that 6 mg of dexamethasone (7–10 days) was less effective in accelerating the recovery and reducing severity markers (CRP, D-dimer, and LDH) than high-dose methylprednisolone (3 days), followed by prednisone (2 weeks) (Pinzón et al., 2021). Moreover, in another clinical study, 60-day mortality was not reduced when high-dose dexamethasone was prescribed in patients with COVID-19-related acute hypoxemic respiratory failure (Bouadma et al., 2022). In another randomized controlled trial, it was found that 28-day mortality was increased in hospitalized patients with COVID-19 pneumonia receiving high doses of dexamethasone (20 mg q. d.), in contrast to patients receiving 6 mg of dexamethasone q. d. (Wu et al., 2022).

On the COVID-19 model in hamsters, dexamethasone prevented inflammation and helped preserve the integrity of the lungs (Wyler et al., 2022). Moreover, in hamsters, dexamethasone did not accelerate the virus replication and diminished the number of inflammatory mediators (Wyler et al., 2022). Clinically, the latter effect is important for preventing pulmonary edema and enhancing gas exchange (Wyler et al., 2022). In SARS-CoV-2-infected rhesus macaques, inhalation of a dose of 0.01 mg/kg nanoDEX (engineered neutrophil nanovesicles to deliver dexamethasone) reduced lung inflammation and preserved their integrity better compared to the intravenous administration of 0.1 mg/kg dexamethasone (Meng et al., 2023). However, in the other hamster COVID-19 model, dexamethasone administration contributed to a decrease in the serum-neutralizing antibody and RBD-specific antibody titers, which led to a minor growth of viral replication (Yuan et al., 2022).

Our data suggest that dexamethasone, at least in our DAD model and dosage regime, did not have any resulted beneficial effect on ARDS pathology. It showed some non-statistically significant effect revealed by histological analysis in the early period of ARDS but failed to show any efficacy according to other evaluation techniques. One may suggest that murine models may be inappropriate for the testing of dexamethasone therapeutic potential as Xu et al. showed that dexamethasone treatment in the dosage of 2.5 mg/kg from days 3 to 14 post-inoculation has no beneficial effect on ARDS in mice caused by the H5N1 virus (Xu et al., 2009).

Tocilizumab was shown to be effective treatment in patients with severe COVID-19 (Xu et al., 2020). It has a positive effect on improving immune damage, lung functional injuries, and arterial oxygen saturation. In addition, it was shown that tocilizumab reduces the number of HDL-1 subfractions of cholesterol, phospholipids, and Apo A2 and increases the levels of LDL-5, HDL-4, IDL, VLDL-1, and VLDL-2, which helps in partially restoring the indicators changed due to coronavirus infection (Meoni et al., 2021). However, in the randomized trial involving hospitalized patients with severe COVID-19 pneumonia, the use of tocilizumab did not result in significantly better clinical status or lower mortality than placebo at 28 days (Rosas et al., 2021). Moreover, in another randomized clinical trial, it was shown that after tocilizumab administration, the risk of intubation or death, disease condition, or time to discontinuation of supplemental oxygen did not significantly change in patients with COVID-19 (Stone et al., 2020). In another clinical trial, it was found that 400 mg of tocilizumab did not improve hypoxemia on days 14 or 28 and did not change ventilator-free survival on day 14 among patients with COVID-19 (Mehta et al., 2021). Finally, the following clinical trial revealed that tocilizumab (8 mg/kg) may elevate mortality, and its administration did not lead to better clinical outcomes at 15 days (Veiga et al., 2021). In a murine model of COVID-19, it was found that the use of interleukin-6 receptor blockers does not change the contents of IL-1 and TNF, although it diminishes neutrophil infiltration (Gu et al., 2020).

We showed that tocilizumab significantly decreased the release of cytokines and chemokines and inhibited mononuclear infiltration during DAD development. Despite several reports claiming effects of tocilizumab in various experimental murine models (Hu et al., 2018; Orabona et al., 2018; Wu et al., 2018; Kamiya et al., 2019), the study by Loka et al. (2020) showed that tocilizumab does not block IL-6 signaling in murine cells. These data may explain the absence of tocilizumab effects on complex injured lung restoration in our study visualized by computed tomography.

COVID-19 may also be treated with leronlimab–a humanized monoclonal antibody to CCR5 (PRO 140), which binds to the extracellular loop 2 domain and the N-terminus of CCR5 (Patterson et al., 2021). Interestingly, healthy *rhesus macaques* receiving 10 mg/kg or 50 mg/kg leronlimab injection had elevated CCR5+CD4^+^ T-cell counts (Chang et al., 2021). Furthermore, this drug was administered subcutaneously to COVID-19 patients on days 0 and 7 of the study, which led to a decrease in the inflammatory mediator IL-6 concentration, recovery of the CD4/CD8 ratio, and a reduction in plasma viremia (pVL) (Patterson et al., 2021). Another case study also showed a decrease in the first and a recovery of the second indicator (Agresti et al., 2021). In “long-term COVID-19,” leronlimab administered at 700 mg s.c. weekly also normalized the immune downmodulation (Gaylis et al., 2022).

Files et al. reported that the CCR2 and CCR5 blocker cenicriviroc reduces the infiltration of monocytes and lymphocytes into the lung tissue (Files et al., 2022) and prevents the replication of SARS-CoV-2 in VeroE6/TMPRSS2 cells by inhibiting virus-dependent cell destruction (EC_50_ = 19 µM) (Okamoto et al., 2020). This effect may not have a direct impact on the virus, but the possible restriction of myeloid-derived suppressor cells may increase the pool of effector lymphoid B and T cells that will exhibit antiviral exposure in an indirect way (Files et al., 2022). In one clinical trial, cenicriviroc (100 and 200 mg) was effective and well-tolerated by HIV-1-infected patients (Thompson et al., 2016). Thus, a growth in the concentration of cenicriviroc contributed to the improvement of virological outcomes (Thompson et al., 2016). In another clinical trial, cenicriviroc (150 mg) ameliorated fibrosis, prevented the progression of steatohepatitis, and diminished the indicators of systemic inflammation in patients with non-alcoholic steatohepatitis (Friedman et al., 2018). In the third clinical trial in patients with the same disease, cenicriviroc (150 mg) also precluded systemic inflammation and fibrosis by reducing C-reactive protein, IL-6, IL-1β, and fibrinogen levels (Ratziu et al., 2020). However, a randomized clinical trial revealed that hospitalized COVID-19 patients treated with cenicriviroc did not recover faster than those patients who received a placebo (O’Halloran et al., 2023). Another low-weight CCR5 antagonist, maraviroc, was also applied for COVID-19 treatment. This drug is quite promising due to its low protein-binding efficiency and high bioavailability (Shamsi et al., 2020). At the same time, maraviroc strongly inhibits SARS-CoV-2 Mpro and suppresses the coronavirus infection development (Shamsi et al., 2020). In “long-term COVID-19,” the combination of maraviroc and pravastatin improved clinical indicators among 18 patients in a case study (Patterson et al., 2023). As of today, two clinical trials (NCT04435522 and NCT04710199) have been completed and two (NCT04441385 and NCT04475991) have been terminated. Nevertheless, in the African green monkey kidney cell model of COVID-19, it was demonstrated that the administration of maraviroc reduces the viral load by precluding membrane fusion, which affects the reproduction and dissemination of the coronavirus (Risner et al., 2022). In the same study, it was revealed that maraviroc suppresses S-protein transport to the extracellular surface of the cell.

Our data uniquely read that the selective CCR5/CXCR3 inhibitor TAK-779 is highly efficient in the prevention of the ARDS in our murine DAD model. Its therapeutic effect is reasoned by a milder course of the inflammatory process in the lungs with early and effective involvement of the affected lung lobe in the systemic gas exchange. These suggestions are confirmed by a low degree of fibrosis and volume loss of the injured lung, decreased mononuclear infiltration, and the rate of density restoration among animals treated with TAK-779. A more favorable Kernogan index observed in this group is as an important quantitative indicator of the capacity of small-circle vessels and, as a consequence, an indicator of the load on the systemic blood flow. Our study indicates that TAK-779 can effectively alleviate DAD and lung collapse in mice by its direct inhibitory effects on inflammation, cytokine release, and immune cell migration. The CXCR3 and CCR5 receptors in humans are actively involved in immune hyperactivation during SARS-CoV-2 infection (Chua et al., 2020). The limitation to our study is that the acute phase of LPS-induced ARDS is 12–24 h, whereas the SARS-CoV-2-induced lung injury reaches its peak in 72–120 h. Thus, in case of COVID-19, TAK-779 may be potentially administrated daily for 4–5 days directly after clinical symptoms of SARS-CoV-2 infection or for the same period in case of the critical disease onset.

## Conclusion

The pathogenesis of the SARS-CoV-2 infection is tightly linked with the cytokine storm, resulting in the enormous release of cytokines and chemokines. Its clinical manifestation, the acute respiratory distress syndrome (ARDS), may be caused by self-sustaining hypersensitivity reactions, leading to lung collapse even after virus clearance. Here, we report that two macrophage inflammatory proteins, namely, MIP-1α/CCL3 and MIP-1β/CCL4, seem to orchestrate mononuclear infiltration into the lungs during diffuse alveolar damage (DAD) in ICR mice—our murine model of ARDS caused by SARS-CoV-2. Inhibition of the C–C chemokine receptor 5 (CCR5)—a parental receptor for MIP-1α and MIP-1β, by the nonpeptide antagonist TAK-779—results in significant amelioration of DAD in terms of reduced mononuclear infiltration into the lung, suppressed cytokine storm, and restored physiology of the affected lung, according to computed tomography data. We finally suggest that targeted inhibition of CCR5 should be further elucidated as a safe and effective approach to overcome severe viral pneumonia in humans.

## Data Availability

The raw data supporting the conclusion of this article will be made available by the authors, without undue reservation.
